# Eventration of Diaphragm with Hiatal Hernia: A Case Report

**Published:** 2012-04-01

**Authors:** Bilal Mirza, Afzal Sheikh

**Affiliations:** Department of Paediatric Surgery, The Children's Hospital and the Institute of Child Health Lahore, Pakistan

**Keywords:** eventration of diaphragm, hiatal hernia

## Abstract

A 25-day-old female baby having eventration of diaphragm associated with a big hiatal hernia is being reported here. This is the second report describing the rare association.

## INTRODUCTION

Hernia, eventration, and agenesis are the main congenital birth defects that affect diaphragm. Congenital diaphragmatic hernia is the frequent defect [1]. These defects usually occur in isolation and rarely coexist. A single report is available regarding the association of eventration and hiatal hernia in human beings [2]. We are reporting the second report on this rare association.

## CASE REPORT

A 25-day-old neonate, weighting 3kg, presented with non-bilious vomiting for 2 weeks and respiratory distress for a week. He was a product of consanguineous marriage and born by spontaneous vaginal delivery with no perinatal problems. On clinical examination the patient was tachypneic (respiratory rate 55/min), with temperature 99F and heart rate of 120/min. Chest auscultation showed reduced air entry on posterior aspects of right lower chest. Chest radiograph was performed that showed right eventration of diaphragm (Fig. 1).

**Figure:**
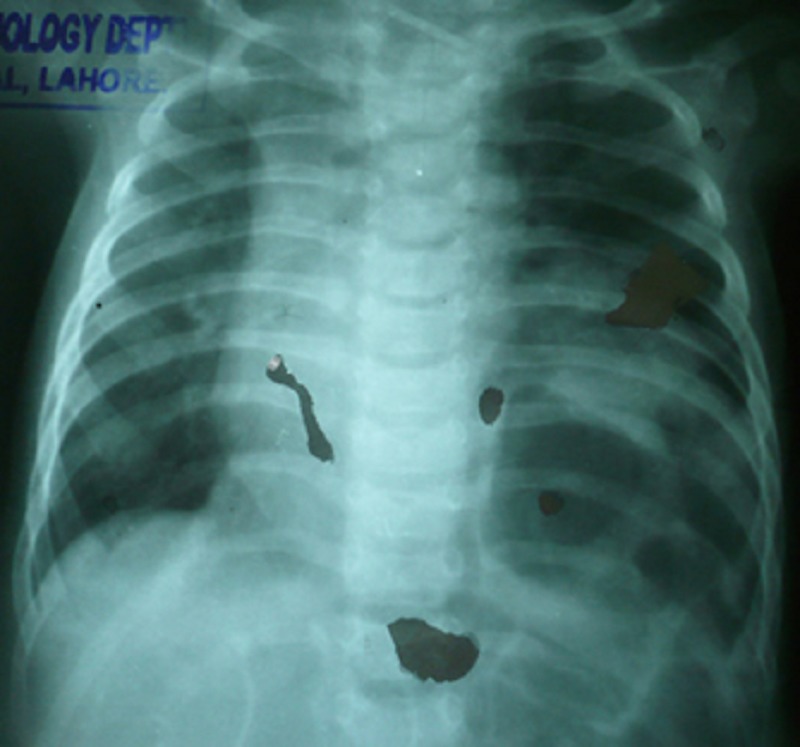
Figure 1: X-ray chest showing eventration of diaphragm.

Patient was stabilized by oxygen inhalation, steam nebulization, and antibiotics. Operation was performed that revealed a small eventration of diaphragm not coinciding with the radiographic delineation. Further exploration revealed absent stomach in the peritoneal cavity which was entirely present in the chest across a big hiatal hernia (Fig. 2,3). The stomach was retrieved back to the peritoneal cavity and hiatal hernia was repaired after excising the hernia sac. Eventration of diaphragm was also repaired. The postoperative recovery was uneventful. Postoperative chest radiograph was indicative of proper repair of the defects. The patient is doing well on follow up.

**Figure:**
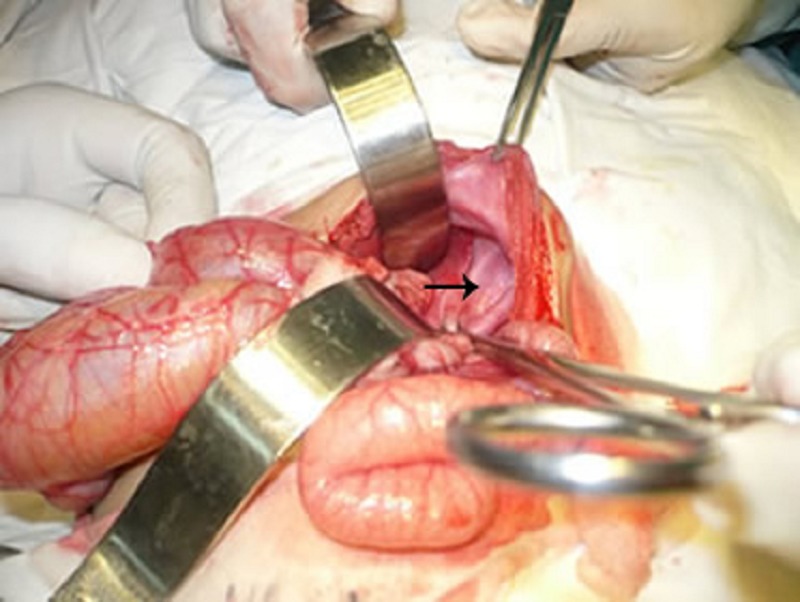
Figure 2: A small eventration.

**Figure:**
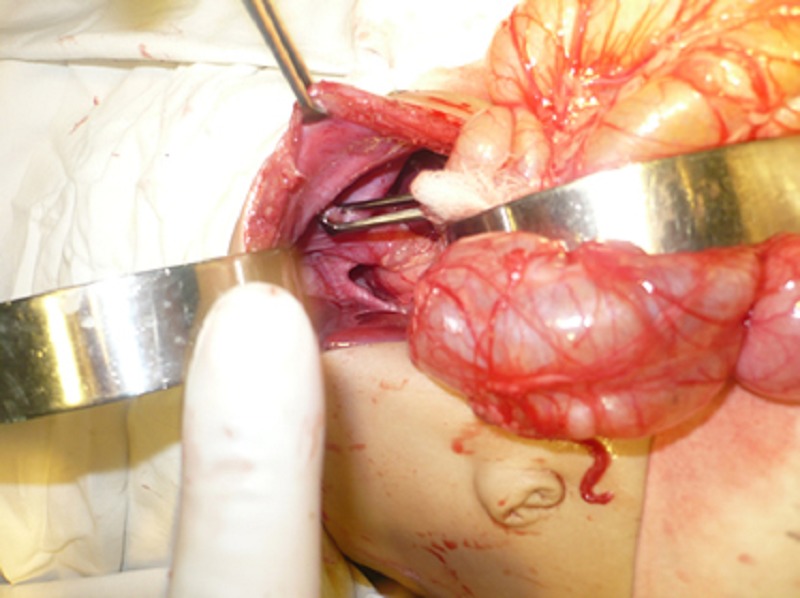
Figure 3: A big hiatal hernia causing herniation of entire stomach into chest.

## DISCUSSION

In the present case the big hiatal hernia was simulating an eventration of left hemi-diaphragm, nevertheless the eventration was also present. Inspection of hiatus is necessary if one finds incommensurately small eventration as to the radiographic delineation. In our case a small eventration was there; on further exploration stomach was not present within the abdomen. Therefore hiatus was inspected and entire stomach was delivered out of the chest, followed by its repair. A 5% rate of fatal complications related to big hiatal hernia are reported; incarceration, gastric perforation and volvulus are the most dreadful ones [3]. In the first report of this rare association, the hiatal hernia was missed during first operation that led to gastric volvulus and thus required an emergent surgery [1]. Therefore we recommend inspecting the hiatus in all types of diaphragmatic repairs in order to avoid sinister complications.

## Footnotes

**Source of Support:** Nil

**Conflict of Interest:** None declared

